# Supplementary material for: Temporal stratification of amyotrophic lateral sclerosis patients using disease progression patterns
Source: Nat Commun. 2024 Jul 8;15:5717. doi: 10.1038/s41467-024-49954-y (PMC11231290; doi:10.1038/s41467-024-49954-y)
Supplement: Supplementary file 1 — Supplementary Information [file 41467_2024_49954_MOESM1_ESM.pdf]

Temporal Stratification of Amyotrophic Lateral Sclerosis Patients using Disease Progression Patterns

Supplementary Material

1 The role of static features in ALS stratification with ClusTric

The ClusTric method enables the inclusion of static features in the identification of disease progression groups. The following supplementary experiments study the impact of incorporating static features (Table 1) for stratification when using three appointments (Section 1.1) and one appointment (Section 1.2).

1.1 Stratification using static features and three appointments

Static features are considered in the similarity matrix by computing 2D patterns through biclustering as explained in the Methods section. Figure S1 shows the progression groups and respective disease progression trajectories found by ClusTric when considering static features and the first three consecutive appointments of patient follow-up. Figure S2 presents the same information when static features are used.

These results elucidate the following findings:

- When considering the inclusion of static patterns together with temporal ones, the best partition according the used criteria leads to three clusters (contrasting with the four obtained without considering static patterns, shown in Fig. 1). This prevents the finding of the Moderate Progressors mainly spinal group.
- When considering only the static patterns, the best partition leads to seven groups, but without coherent disease progressions, highlighting that static patterns reflect the high patient heterogeneity but are insufficient to capture disease progression.

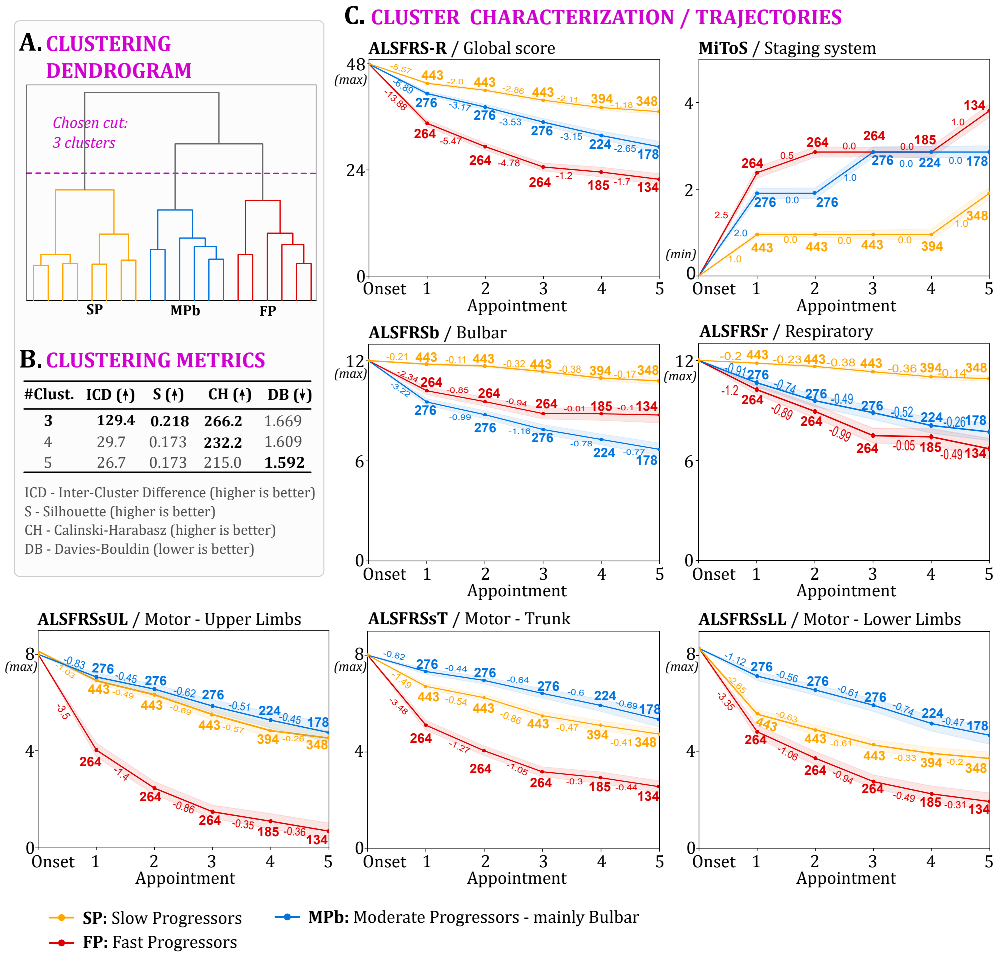

#Clust.	ICD (↑)	S (↑)	CH (↑)	DB (↓)
**3**	**129.4**	**0.218**	**266.2**	1.669
4	29.7	0.173	**232.2**	1.609
5	26.7	0.173	215.0	**1.592**

Fig. S1: **Experiment with ClusTric using static features and first 3 consecutive appointments - Cluster analysis and characterization on Lisbon ALS cohort.** (A) Dendrogram resulting from ClusTric; (B) corresponding evaluation scores obtained with 3, 4 and 5 clusters; and (C) average temporal feature trajectories (lines) and 95% confidence intervals (shades around lines). The numbers next to each point in the trajectories represent the number of patients in the <cluster,appointment> set, whereas the numbers between consecutive appointments indicate the average slope between consecutive measurements in a cluster. Source data are provided as a Source Data file.

A. CLUSTERING DENDROGRAM

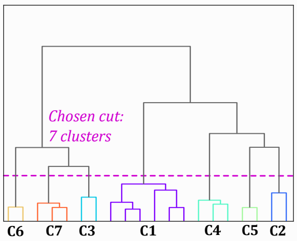

B. CLUSTERING METRICS

#Clust.	ICD (↑)	S (↑)	CH (↑)	DB (↓)
3	**102.9**	0.509	755.8	0.847
4	45.7	0.520	802.7	0.740
5	0.9	0.600	893.0	0.637
6	63.0	0.621	1186.8	0.673
**7**	58.7	**0.681**	**1451.6**	**0.622**

ICD - Inter-Cluster Difference (higher is better)
S - Silhouette (higher is better)
CH - Calinski-Harabasz (higher is better)
DB - Davies-Bouldin (lower is better)

C. CLUSTER CHARACTERIZATION / TRAJECTORIES

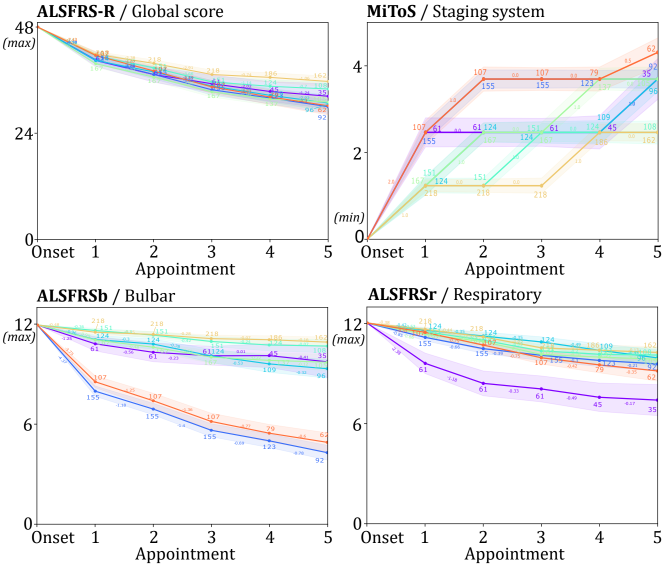

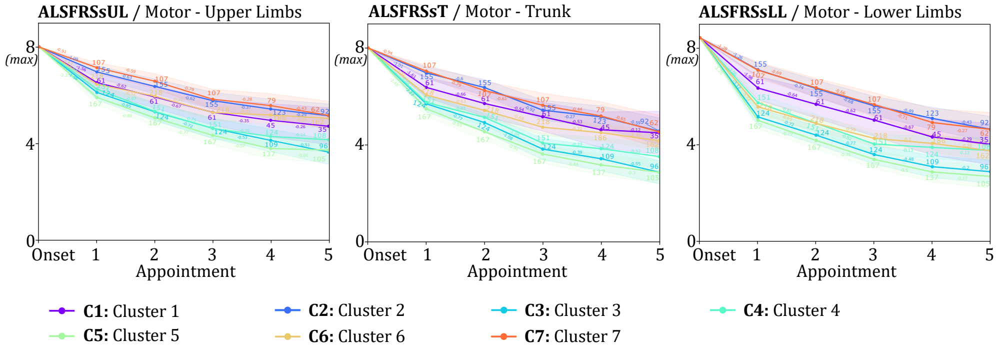

Fig. S2: **Experiment with ClusTric using ONLY static features - Cluster analysis and characterization on Lisbon ALS cohort.** (A) Dendrogram resulting from ClusTric; (B) corresponding evaluation scores obtained with 3-7 clusters; and (C) average temporal feature trajectories (lines) and 95% confidence intervals (shades around lines). The numbers next to each point in the trajectories represent the number of patients in the <cluster,appointment> set, whereas the numbers between consecutive appointments indicate the average slope between consecutive measurements in a cluster. Source data are provided as a Source Data file.

1.2 Stratification using first appointment

We performed experiments considering the features usually assessed at patients' follow-up (Table 2) with and without the static features (Table 1). Figure S3 presents the progression groups and respective disease progression trajectories found by ClusTric without static features, while Figure S4 depicts the same information but with static features. From these results, we highlight the following findings:

- ClusTric found three groups when considering the ALSFRS scores at first appointment, with disjoint temporal coherent trajectories.
- The inclusion of static features proved again the higher heterogeneity of patients when compared with the less heterogeneity found by the ALSFRS functional scale.

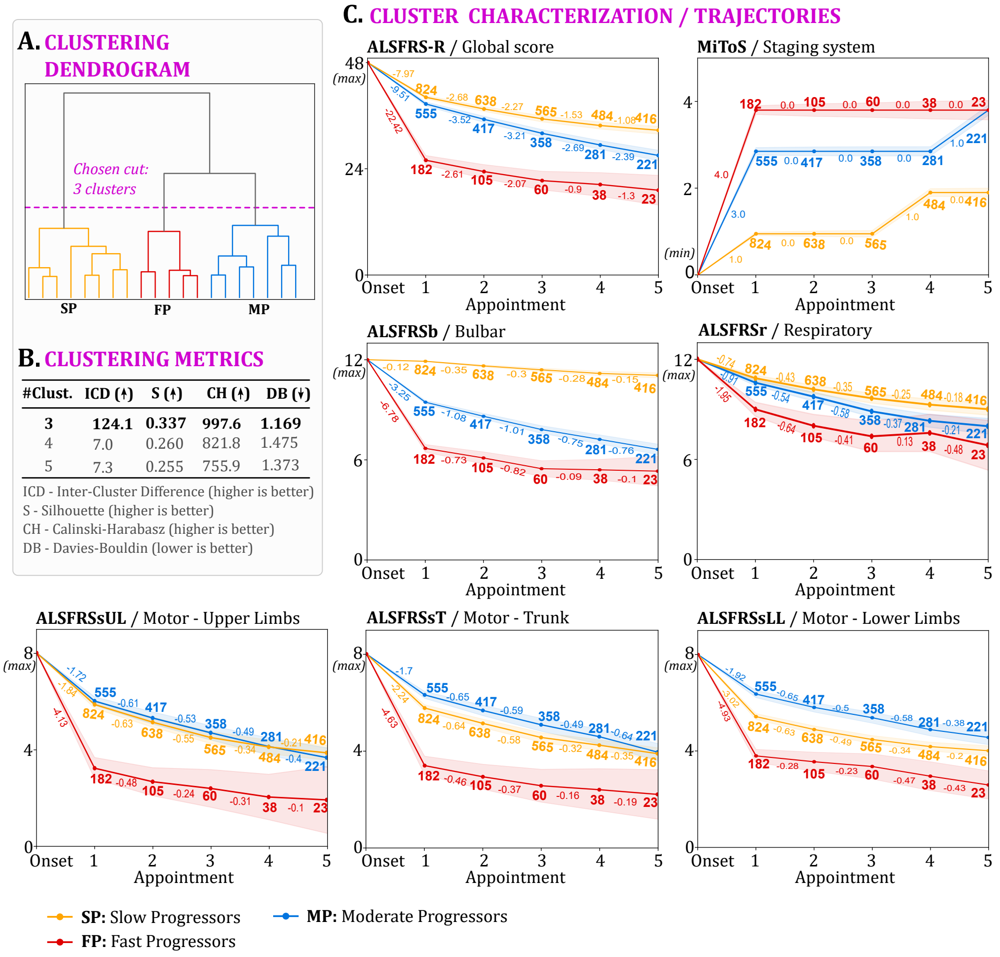

Fig. S3: **Experiment with ClusTric using ONLY first appointment data - Cluster analysis and characterization on Lisbon ALS cohort.** (A) Dendrogram resulting from ClusTric; (B) corresponding evaluation scores obtained with 3, 4 and 5 clusters; and (C) average temporal feature trajectories (lines) and 95% confidence intervals (shades around lines). The numbers next to each point in the trajectories represent the number of patients in the <cluster,appointment> set, whereas the numbers between consecutive appointments indicate the average slope between consecutive measurements in a cluster. Source data are provided as a Source Data file

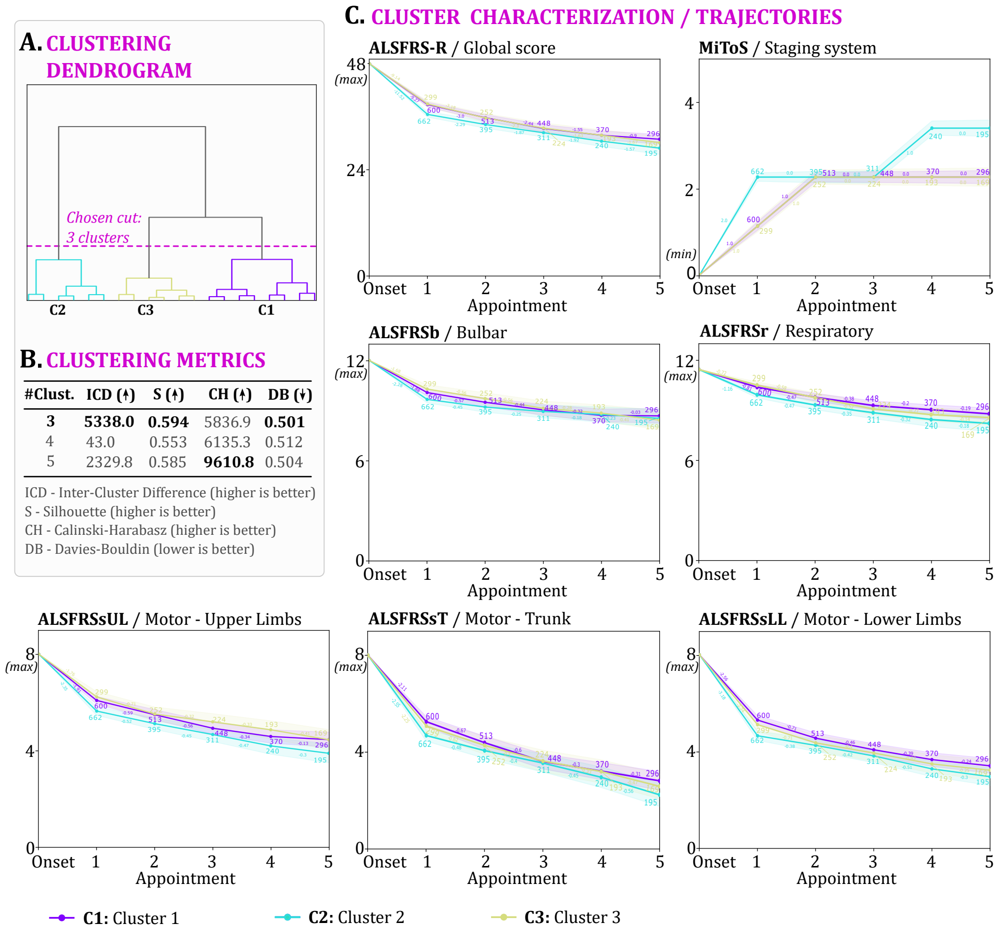

#Clust.	ICD (↑)	S (↑)	CH (↑)	DB (↓)
3	**5338.0**	**0.594**	5836.9	**0.501**
4	43.0	0.553	6135.3	0.512
5	2329.8	0.585	**9610.8**	0.504

Fig. S4: **Experiment with ClusTric using static features and first appointment data - Cluster analysis and characterization on Lisbon ALS cohort.** (A) Dendrogram resulting from ClusTric; (B) corresponding evaluation scores obtained with 3, 4 and 5 clusters; and (C) average temporal feature trajectories (lines) and 95% confidence intervals (shades around lines). The numbers next to each point in the trajectories represent the number of patients in the <cluster,appointment> set, whereas the numbers between consecutive appointments indicate the average slope between consecutive measurements in a cluster. Source data are provided as a Source Data file.

2 Other Supplementary Material

Table S1: **Characterization of the population used in the case study based on the temporal data.** Characterization of the population in the PRO-ACT dataset and the Lisbon ALS Clinic dataset based on measurements from the 1st and 3rd visits. The features are described using Median, Interquartile Range (IQR), Average, and Standard deviation (Std).

	**PRO-ACT** n = 3880		**Lisbon ALS** n = 983	
	1st visit	3rd visit	1st visit	3rd visit
**ALSFRS-R**				
Median, IQR	39, 35 - 42	37, 32 - 41	41, 37 - 44	34, 28 - 40
Average, Std	37.91, 5.30	35.61, 6.76	39.83, 5.87	33, 9.07
**ALSFRSb**				
Median, IQR	11, 9 - 12	11, 9 - 12	12, 9 - 12	10, 7 - 12
Average, Std	10.27, 2.16	9.85, 2.59	11.38, 2.23	9.10, 3.36
**ALSFRSsUL**				
Median, IQR	6, 5 - 7	6, 4 - 7	7, 6 - 8	5, 3 - 7
Average, Std	5.80, 1.92	5.30, 2.20	6.36, 1.86	4.92, 2.59
**ALSFRSsLL**				
Median, IQR	4, 3 - 7	4, 3 - 6	6, 4 - 8	4, 2 - 6
Average, Std	4.77, 2.22	4.28, 2.30	5.59, 2.11	4.17, 2.51
**ALSFRSsT**				
Median, IQR	6, 5 - 7	5, 4 - 7	7, 5 - 8	5, 3 - 7
Average, Std	5.67, 1.73	5.11, 2.00	6.16, 1.85	4.61, 2.57
**ALSFRSr**				
Median, IQR	12, 11 - 12	12, 11 - 12	12, 11 - 12	11, 9 - 12
Average, Std	11.38, 1.14	11.06, 1.64	11.33, 1.30	10.20, 2.27

Table S2: **Disease onset and 1st visit characterization.** Characterization of each PRO-ACT cluster according to some features at disease onset and at 1st visit, and the significance level of each feature. Categorical features (sex) are represented by the number and percentage of patients presenting that characteristic within the cluster. Continuous features (age at onset, diagnostic delay, BMI, ALSFRS-R at 1st visit, Slope of ALSFRS-R at 6 months) are represented by mean value (first row for each variable) and 95% confidence interval (second row). The statistical tests used for data analysis were the Chi-square two-sided test for categorical features, and the non-parametric Kruskal-Wallis two-sided test for non-normal distribution continuous features with the Bonferroni correction.

**Characteristic**	**Overall**<br>N = 3880	**Cluster SP**<br>N = 1377	**Cluster MPb**<br>N = 448	**Cluster MPs**<br>N = 878	**Cluster FP**<br>N = 1177	**p-value**
**Male**	2460<br>63.4%	1025<br>74.4%	196<br>43.8%	510<br>58.1%	729<br>61.9%	3.7×10^{-34}
**Age at onset**<br>(years)	56.20<br>[55.81 ; 56.59]	55.31<br>[54.29 ; 56.32]	61.35<br>[59.66 ; 63.03]	57.40<br>[56.14 ; 58.67]	54.87<br>[53.57 ; 56.16]	0<br>SP = FP
**Diagnostic Delay**<br>(months)	11.30<br>[11.01; 11.59]	10.19<br>[9.53 ; 10.85]	10.03<br>[8.84 ; 11.22]	11.90<br>[10.83 ; 12.97]	9.64<br>[8.86 ; 10.42]	1.2×10^{-4}<br>MPs $\neq$ {SP, FP}
**BMI**	26.63<br>[26.39 ; 26.87]	26.49<br>[26.06 ; 26.91]	25.57<br>[24.76 ; 26.38]	26.75<br>[26.08 ; 27.41]	26.81<br>[26.17 ; 27.45]	2.4×10^{-4}<br>MPb $\neq$ {SP,MPs, FP}
**ALSFRS-R at 1st visit**	37.91<br>[37.74 ; 38.08]	42.71<br>[42.46 ; 42.95]	35.60<br>[34.85 ; 36.36]	39.08<br>[38.79 ; 39.37]	32.74<br>[32.28 ; 33.20]	0<br>SP $\neq$ MPb $\neq$ MPs $\neq$ FP
**Slope of ALSFRS-R at 6 months**	-0.96<br>[-0.99 ; -0.93]	-0.80<br>[-0.85 ; -0.76]	-1.31<br>[-1.42 ; -1.21]	-0.76<br>[-0.82 ; -0.70]	-1.17<br>[-1.24 ; -1.11]	0<br>SP = MPs and MPb = FP

Table S3: **Pairwise comparisons of survival curves of Figure 4.** Log-rank test was used to test differences between pairwise survival curves for MoGP and ClusTric.

	MoGP		ClusTric
G1 vs G2	0.524	SP vs MPb	$\mathbf{1.0 \times 10^{-24}}$
G1 vs G3	**0.002**	SP vs MPs	$\mathbf{1.8 \times 10^{-16}}$
G1 vs G4	0.223	SP vs FP	$\mathbf{3.9 \times 10^{-22}}$
G2 vs G3	$\mathbf{5.4 \times 10^{-4}}$	MPb vs MPs	0.955
G2 vs G4	0.335	MPb vs FP	**0.001**
G3 vs G4	$\mathbf{7.0 \times 10^{-5}}$	MPs vs FP	**0.007**

Table S4: **Pairwise comparisons of survival curves of Figure 5.** Log-rank test was used to test differences between pairwise survival curves of patients coming from SP group.

	ClusTric
SP vs MPb	1.5×10^{-16}
SP vs MPs	1.3×10^{-8}
MPb vs MPs	0.025
