# Supplementary material for: Temporal stratification of amyotrophic lateral sclerosis patients using disease progression patterns
Source: Nat Commun. 2024 Jul 8;15:5717. doi: 10.1038/s41467-024-49954-y (PMC11231290; doi:10.1038/s41467-024-49954-y)
Supplement: Supplementary file 2 — Reporting Summary [file 41467_2024_49954_MOESM2_ESM.pdf]

nature portfolio

Corresponding author(s): Diogo F. Soares, Helena Aidos

Last updated by author(s): Jun 5, 2024

Reporting Summary

Nature Portfolio wishes to improve the reproducibility of the work that we publish. This form provides structure for consistency and transparency in reporting. For further information on Nature Portfolio policies, see our Editorial Policies and the Editorial Policy Checklist.

Statistics

For all statistical analyses, confirm that the following items are present in the figure legend, table legend, main text, or Methods section.

n/a	Confirmed	
☐	☒	The exact sample size (*n*) for each experimental group/condition, given as a discrete number and unit of measurement
☒	☐	A statement on whether measurements were taken from distinct samples or whether the same sample was measured repeatedly
☐	☒	The statistical test(s) used AND whether they are one- or two-sided<br>*Only common tests should be described solely by name; describe more complex techniques in the Methods section.*
☒	☐	A description of all covariates tested
☐	☒	A description of any assumptions or corrections, such as tests of normality and adjustment for multiple comparisons
☐	☒	A full description of the statistical parameters including central tendency (e.g. means) or other basic estimates (e.g. regression coefficient) AND variation (e.g. standard deviation) or associated estimates of uncertainty (e.g. confidence intervals)
☐	☒	For null hypothesis testing, the test statistic (e.g. *F*, *t*, *r*) with confidence intervals, effect sizes, degrees of freedom and *P* value noted<br>*Give P values as exact values whenever suitable.*
☒	☐	For Bayesian analysis, information on the choice of priors and Markov chain Monte Carlo settings
☐	☒	For hierarchical and complex designs, identification of the appropriate level for tests and full reporting of outcomes
☒	☐	Estimates of effect sizes (e.g. Cohen's *d*, Pearson's *r*), indicating how they were calculated

Our web collection on statistics for biologists contains articles on many of the points above.

Software and code

Policy information about availability of computer code

Data collection	No software was used.
Data analysis	All the code needed to run the method proposed in this work are available at: https://github.com/LxMLearners/ClusTric and https://doi.org/10.5281/zenodo.11471491<br><br>Python: 3.10.11<br><br>Versions of packages:<br>numpy: 1.23.4<br>pandas: 1.5.1<br>matplotlib: 3.6.3<br>sklearn: 1.2.2<br>seaborn: 0.12.2<br>scipy: 1.10.0<br>pacmap: 0.7.0<br>sortedcontainers: 2.4.0<br>yaml: 6.0<br><br>Further analysis was performed using SPSS v.29

For manuscripts utilizing custom algorithms or software that are central to the research but not yet described in published literature, software must be made available to editors and reviewers. We strongly encourage code deposition in a community repository (e.g. GitHub). See the Nature Portfolio guidelines for submitting code & software for further information.

Data

Policy information about availability of data

All manuscripts must include a data availability statement. This statement should provide the following information, where applicable:

- Accession codes, unique identifiers, or web links for publicly available datasets
- A description of any restrictions on data availability
- For clinical datasets or third party data, please ensure that the statement adheres to our policy

The Lisbon ALS Clinic dataset analysed during the current study is available under restricted access to ensure patients' rights to privacy and anonymity; access can be obtained by contacting Prof. Mamede de Carvalho (Instituto de Medicina Molecular – Faculdade de Medicina Universidade de Lisboa; expected response time: 1 month).
PRO-ACT database is available for download upon registration in \url{https://alsdatabse.org}

Research involving human participants, their data, or biological material

Policy information about studies with human participants or human data. See also policy information about sex, gender (identity/presentation), and sexual orientation and race, ethnicity and racism.

Reporting on sex and gender	Sex was considered in the study design as it is an important attribute to be considered in the disease study. No individual-level data was shared. The study included 545 males and 438 females.<br>Gender was neither considered nor collected because it is irrelevant to the study.
Reporting on race, ethnicity, or other socially relevant groupings	No socially constructed or socially relevant categorization variables were used as they are irrelevant to our study. No race, ethnicity, or other socially relevant grouping was reported.
Population characteristics	Relevant characterization of the population used in the case study is described in Table 1 of the manuscript.
Recruitment	All ALS Patients who have been regularly monitored at the local ALS clinic since 1995 were initially included. Exclusion criteria: Patients with fewer than three appointments.<br>No recruitment biases are expected to have occurred.
Ethics oversight	Comissão de Ética do Centro Académico de Medicina de Lisboa (CHLN/FMUL/IMM)

Note that full information on the approval of the study protocol must also be provided in the manuscript.

Field-specific reporting

Please select the one below that is the best fit for your research. If you are not sure, read the appropriate sections before making your selection.

☒ Life sciences ☐ Behavioural & social sciences ☐ Ecological, evolutionary & environmental sciences

For a reference copy of the document with all sections, see nature.com/documents/nr-reporting-summary-flat.pdf

Life sciences study design

All studies must disclose on these points even when the disclosure is negative.

Sample size	We conducted our study using the Lisbon ALS clinic dataset, which consists of Electronic Health Records from ALS Patients who have been regularly monitored at the local ALS clinic since 1995. The used dataset was last updated in May 2023 and includes 1677 patients.
Data exclusions	Patients with fewer than three appointments were excluded from the analysis, resulting in a total of 983 patients in the Lisbon ALS Clinic dataset and 3880 in the PRO-ACT dataset
Replication	We conducted extensive analysis to evaluate the robustness of our results. These included comparing model results across clinical cohorts. All the results are reported in the manuscript. The number of datasets used in this study was determined by the availability of ALS data. Using both datasets ensures that our findings are clinically relevant and broadly applicable. Experiments were performed once and independently for each dataset.
Randomization	As our study does not involve a comparison between a treatment group and a control group, the concept of randomization is not applicable. Instead, to address covariates, we incorporated two extensive clinical cohorts. This allowed us to assess the performance of the model across prevalent sources of covariates in clinical data, including variations in the rate of progression within each cohort, the frequency of clinical visits, the size of the clinical study, and the diversity of clinical sites.
Blinding	The Lisbon Clinical Dataset is composed of anonymized clinical data from patients that is not blinded to any particular therapy, although no

Blinding	therapy information is used in this work in any way. PRO-ACT is a large database that aggregates anonymized clinical trial data, without disclosing explicit blinding information.

Reporting for specific materials, systems and methods

We require information from authors about some types of materials, experimental systems and methods used in many studies. Here, indicate whether each material, system or method listed is relevant to your study. If you are not sure if a list item applies to your research, read the appropriate section before selecting a response.

Materials & experimental systems

n/a	Involved in the study	
☒	☐	Antibodies
☒	☐	Eukaryotic cell lines
☒	☐	Palaeontology and archaeology
☒	☐	Animals and other organisms
☐	☒	Clinical data
☒	☐	Dual use research of concern
☒	☐	Plants

Methods

n/a	Involved in the study	
☒	☐	ChIP-seq
☒	☐	Flow cytometry
☒	☐	MRI-based neuroimaging

Clinical data

Policy information about clinical studies

All manuscripts should comply with the ICMJE guidelines for publication of clinical research and a completed CONSORT checklist must be included with all submissions.

Clinical trial registration	Lisbon ALS Clinic dataset does not comprise clinical trials.
Study protocol	Lisbon ALS Clinic dataset does not comprise clinical trials.
Data collection	ALS Patients have been regularly monitored at the local ALS clinic (at Centro Hospitalar de Lisboa Norte) since 1995 until May 2023.
Outcomes	A data-driven methodology that stratifies patients based on temporal data and without any linearity assumption to find relevant disease subgroups. We assess each subgroup's mean trajectories of key features and characterize them based on the static features. We complement this characterization with a survival analysis of each progression group considering an 8-year follow-up. Additionally, we study how the patients' disease progression evolves in 6 months of follow-up.

Plants

Seed stocks	*Report on the source of all seed stocks or other plant material used. If applicable, state the seed stock centre and catalogue number. If plant specimens were collected from the field, describe the collection location, date and sampling procedures.*
Novel plant genotypes	*Describe the methods by which all novel plant genotypes were produced. This includes those generated by transgenic approaches, gene editing, chemical/radiation-based mutagenesis and hybridization. For transgenic lines, describe the transformation method, the number of independent lines analyzed and the generation upon which experiments were performed. For gene-edited lines, describe the editor used, the endogenous sequence targeted for editing, the targeting guide RNA sequence (if applicable) and how the editor was applied.*
Authentication	*Describe any authentication procedures for each seed stock used or novel genotype generated. Describe any experiments used to assess the effect of a mutation and, where applicable, how potential secondary effects (e.g. second site T-DNA insertions, mosiacism, off-target gene editing) were examined.*
